# A novel approach to the analysis of human growth

**DOI:** 10.1186/1742-4682-9-17

**Published:** 2012-05-17

**Authors:** Antonio S Gliozzi, Caterina Guiot, Pier Paolo Delsanto, Dan A Iordache

**Affiliations:** 1 CNISM, Politecnico of Torino, Physics Department, Corso Duca degli Abruzzi 24, 10129, Torino, Italy; 2 Department of Neuroscience, Università di Torino, Corso Raffaello, Torino, Italy; 3 Physics Department, University “Politehnica” of Bucharest, Splaiul Independentei 313, Bucharest, Romania

## Abstract

**Objectives:**

Several formulations have been proposed in order to model human growth from birth to maturity. They are usually based on “ad hoc” heuristic assumptions. In the present contribution we adopt, as an alternative, a completely general (interdisciplinary) approach, based on the formalism of the Phenomenological Universalities (PUN).

**Methods:**

The main PUN class investigated to date, i.e. UN, can only account for the overall growth pattern. For a realistic description it is necessary to add to it one or more “spurts”, as expected on biological grounds, due to the stimulation of growth and sex hormones.

**Results:**

A new PUN class (*UN* + *FM*) is generated and shown to be able to provide excellent agreement with standard auxological datasets. The accuracy of the fitting and reliability of the model suggest applications both at the diagnostic and therapeutic level.

**Conclusions:**

The developed formalism can be suitably related to the biological description of bone plate growth under selective hormonal stimulation on the bone epiphysis; i.e., the additional increase of stature is the “macroscopic” response to a well defined biological signal.

## Background

Growth is an extremely complex and non-linear biological process, driven by hormonal mechanisms and characterized by an intrinsic variability reflecting environmental and genetic influences and individual adaptive responses.

Individual human growth has been widely investigated [1-3]. In his brief history of human growth dynamics, Wales [4] reports that periodic accelerations and decelerations in growth had already been observed in 1777 by Montbeillard in his child, while seasonal and diurnal changes were detected by various authors during the 1960s and in later years [5]. More recently, growth variability has been analyzed in detail leading to the concept of “saltatory growth” [6]. The occurrence of “spurts” or discontinuities in longitudinal growth is now known to occur over small time intervals, and may therefore be detected only by using appropriate time scales.

When observations are made over infrequently collected data in “longitudinal” studies, or when growth charts are produced as “transversal” datasets, combining large numbers of data from a given homogeneous population (e.g. in terms of geographical area and of temporal range), growth variability is largely missed. Nevertheless the median curve from a growth chart usually exhibits spurts at mid-childhood (around 6–7 yr in both sexes) and at puberty (around 12 yr for females and 14 yr for males). Growth charts are very helpful as reference tools for pediatricians, in order to monitor individual growth and provide therapeutic interventions when children’s height is well below median values.

Similarly, animal growth has been shown to follow irregular “saltatory” patterns [7], which again are barely observable in studies based on infrequently collected data over large populations. In fact, at a low resolution time-scale, most animal species exhibit a very regular mass (*m*, or weight) growth pattern, characterized by an initial exponential growth and a progressive decrease of the growth rate *a*(*t*), until a final saturation value, the carrying capacity *M*, is reached. Such “universal” behaviour has been analyzed by West et al. [8,9], who showed that by renormalizing the time by the effective growth rate and considering as dependent variable the ratio (*m*/*M*)^0.25^, most species share the same growth curve. West and collaborators have also investigated the dependence of the exponent value on the fractal nature of tissue perfusion, and the relation between the carrying capacity and some basic biological parameters, such as the cellular mass and metabolic rate of the species. For related literature, see [10-13].

For what concern the biological mechanisms underlying human longitudinal growth, a detailed explanation is still missing, and will probably entail multifactorial contributions to the main effect of selective hormonal stimulation on bone epiphyses. Basically, bone epiphyses are the regions where chondrocyte proliferation in the growth plate occurs; these cells can be seen as “stem-like cells”, whose growth is stimulated, at some specific point, by specific hormones [14]. The most important of these hormones, i.e. the growth hormone GH, is secreted by the adenohypophysis and is controlled by the hypothalamus by means of various GH-stimulating mediators, e.g. GHRH (Growth Hormone Releasing Hormone) and GHR-IH (Growth Hormone Release-Inhibiting Hormone). Sleep, physical training, traumata and nutritional conditions are known to stimulate secretion of GH.

The GH effect on bone growth is known to be mediated by specific receptors, such as the IGF-I (Insulin-like growth factor) receptor [15]. Sex hormones (SH, mainly estrogens, which in both boys and girls stimulate GH and IGF-I secretion) are also known to affect the endochondrial bones. Initially they induce a rapid growth in cartilage, producing the growth peak, then cartilages are calcified and height growth is blocked [16]. Besides constitutional primitive and/or familial causes, any impairment in the production/effectiveness of such hormones may be responsible for pathological short stature [17,18] (and can be counteracted with hormonal therapies, see Ranke et al. [19]). It would therefore be valuable to obtain direct indications relating the growth “spurts” and the biological effects of hormones in order to derive therapeutic guidelines. Such hormonal influence on the basic mechanisms of growth is fully in agreement with West’s assumption that the saturation value (or carrying capacity) is related to the cellular metabolic rate.

With regard to human growth description, many models have been proposed to date, mainly based on heuristic assumptions. A number of mathematical functions have been proposed, starting from the linear-logarithmic model [20,21] and the first exponent function model [22]. The logistic model was subsequently applied to human growth by Ozaki [23] and by Nelder [24]. The Gompertz function [25] was used to describe the height of 24 male and 24 female subjects by Deming [26]. In order to obtain a more realistic description, Thissen et al. [27] derived the double logistic function and, for a higher fitting accuracy, Bock and Thissen [28] proposed the triple logistic function. Similarly, the Jolicoeur model uses three terms [29], while Preece and Baines only use two [30], with a total of 5 free parameters. For a review, see [31,32].

The goal of the present paper is to propose an alternative method for the fitting and modelling of human growth charts and individual (longitudinal) growth curves, based on the formalism of the Phenomenological Universalities (PUN). The PUN approach was inspired by casual observation of striking similarities between growth curves (and other phenomenologies) in different and independent fields and contexts, such as in Elastodynamics, Physics, Biology and Economics. It is explained in some detail in the next Section. In the succeeding section the problem of the exogenous vs. endogenous nature of the spurts is discussed and a specific formalism for their treatment is proposed. In the final section, several datasets of great auxological relevance are analysed in order to assess the applicability of the proposed approach and the validity and significance of the results.

## The PUN approach

Given a set of experimental data, the first thing that one may want to do, in order to extract from it as much information as possible, is to find a suitable fitting function. As a next step it may be desirable to construct a model out of it. For this purpose one should restrict one’s attention to the raw data and analyse them independently of the field of application. Such an unbiased procedure may be provided by the Phenomenological Universalities (PUN) approach, recently proposed by P.P. Delsanto and collaborators [33,34] and applied to a wide range of topics (auxology [35], tumor growth [36,37], nonlinear elasticity [38], and others [39-41]).

In order to describe the PUN methodology from an applicative point of view, let us start with the first order nonlinear growth differential equation

(1) $$\frac{dy}{dt}=a(y)y\text{,}$$

where *y*(*t*) represents the variable of interest (e.g. the height of the children) and *a*(*y*) the (unknown) growth rate. Equivalently, Eq.(1) may be written as

(2) $$\frac{dz}{dt}=a(z),$$

where *z* = 1n(*y*). To integrate Eq.(1), or Eq.(2), it is necessary to make some assumption about the rate *a*, e.g. assuming that the time derivative of the growth rate or acceleration *b* is given by an expansion in *a*, i.e.

(3) $$b=\sum_{i=1}^{N}c_{i}a^{i}=\beta a+\gamma a^{2}+\cdot \cdot \cdot \cdot$$

E.g. for *N* = 2 we have, in addition to the linear term *βa*, also the quadratic term *γa*^2^.

We call UN the class generated by the solution of the coupled differential equations (2) or (1) and (3), when in the latter only the first *N* terms are considered. The functions *y*(*t*) that one obtains for the first UN classes (*N* = 0, 1, 2) have a very wide range of applications. In fact:

1. for *N* = 0, i.e. U0, *b* = 0; *y*(*t*) represents a self-catalytic growth function. By integrating over the two ODEs, Eq. (3) and Eq. (1), we obtain

(4) $$y(t)=y_{0}exp(a_{0}t),$$

where *a*_0_ = *a*(*t* = 0) is the initial growth rate. Here and in the following we normalize the variable *y*(*t*), so that *y*(0) = 1.

2. for *N* = 1, i.e. U1, *b* = *βa* and

(5) $$y(t)=y_{0}exp\left[a_{0}/\beta \left(exp(\beta t)-1\right)\right]\text{.}$$

Equation (5) represents the Gompertz law [25], which has been extensively used in all kinds of growth problems for almost two centuries. The parameter *β* represents a retardation factor, which may be defined as $\beta =-a_{0}/z_{\infty }$, where $z_{\infty }$ is the asymptotic value of *z*(*t*).

3. for *N* = 2, i.e. U2, $b=\beta a+\gamma a^{2}$ and

(6) $$y(t)=y_{0}{\left[1+a_{0}\gamma /\beta \left(1-exp(\beta t)\right)\right]}^{-1/\gamma }\text{,}$$

which yields a generalization of West’s law [8,9,42]. Here *γ* = *p* − 1, where *p* is the growth exponent [33].

## Exogenous vs. endogenous growth processes

The formalism of the previous section is perfectly suitable to describe the evolution of an endogenous system, i.e. one in which all the “seeds” of growth are already present at the initial time (*t* = 0). There are, however, situations in which the “rules of the game” change, suddenly or slowly in a way that was not implicitly foreseeable at the time *t* = 0, possibly as a consequence of “forces” external to the system: we shall call these processes “exogenous”.

It is easy to find examples of exogenous processes in all scientific fields: e.g., a severe sickness or accident (such as the loss of a limb) during childhood, or the inception of angiogenesis in oncology, or a change of state in a thermodynamic process, or a “September 11” for the NYSE or OTC, etc. If the disruption of the system takes place at a well defined time *t*_1_, then the PUN formalism may be applied to both periods (before and after *t*_1_) and the change in the PUN parameters may be used to characterize quantitatively the variation which has occurred. If the change is sudden but its precise timing is not known, then *t*_1_ may be used as an adjustable parameter, in order to pinpoint the precise time of the perturbing event.

However, the distinction between exogenous and endogenous processes may be rather subtle. E.g., in the case of human development we can very well theorize that all the “seeds” are present in the DNA of the embryonic cells, so that the whole process is, at least implicitly, endogenous and a unique PUN curve should describe the entirety of perinatal growth (i.e. both before and after birth). In fact this turns out to be correct, at least as a first approximation [43]. Likewise for the growth spurts mentioned in the Background, which occur both at mid-childhood and at a pre-puberal age due to hormonal stimulation.

A more accurate description of the human growth process can, however, be achieved by an alternative treatment, in which we superimpose on an overall U1 or U2 model one or more “mathematical spurts”, modelled as small additional corrections that are also to be described in the framework of a UN class. The resulting formalism is briefly described in the following and will be adopted in the remainder of this paper.

Let us then assume that the overall growth curve can be fitted, up to a certain level of approximation, by Eq. (5). We have restricted our treatment to the class U1 in order to limit the number of “free” parameters of the model, since the corresponding results are already quite satisfactory (see next section); the improvements achievable with U2 or U3 would be only marginal. It should be noted that, although in Eq. (5) there are three parameters, only one (*β*) should be considered as a “free” parameter, since *y*_0_ and *a*_0_ may be directly evaluated from the first few data.

We then superimpose (i.e. we add) on Eq. (5) a small number *M* (usually one or two) of “spurts”, each given by

(7) $$\Delta y_{m}(t)=G(t_{m},\sigma_{m},t)y_{0m}exp\left\{\left(\frac{a_{m}}{\beta_{m}}\right)\left[exp\left(\beta_{m}(t-t_{m})\right)-1\right]\right\}\text{,}$$

where again we have restricted ourselves to the class U1. In Eq. (7) *y*_0*m*_ is the amplitude of the spurt at *t* = *t*_*m*_, and *G*(*t*_*m*_, *σ*_*m*_, *t*) is the Gauss cumulative function with average value *t*_*m*_ and standard deviation *σ*_*m*_, defined by

(8) $$G(t_{m},\sigma_{m},t)=\frac{1}{\sqrt{2\pi }}\int_{-\infty }^{t}e^{-\frac{{\left(\tau -t_{m}\right)}^{2}}{2\sigma_{m}^{2}}}d\tau$$

A special case of the Gauss cumulative function is the Heaviside function

(9) $$H(t_{m},t)=G(t_{m}\text{,0,}t)=\{\begin{array}{ll} 0 & \;if\;t\leq t_{m};\\ 1 & \;if\;t>t_{m}\text{.} \end{array}$$

The corresponding PUN classes will be called (*U*1 + *GM*) and (*U*1 + *HM*), respectively, with *M* = 1, 2, … being the number of spurts assumed. Obviously, since we are dealing with a steadily evolving process, results will be better with the former class (*U*1 + *GM*), although the latter may be preferable when it is important to decrease the number of free parameters. A good compromise may be to adopt a class, which we shall call (*U*1 + *FM*), which is identical to (*U*1 + *GM*) except that the *a*_0*m*_ parameters for the spurts are assumed to be equal to the U1 parameter *a*_0_. Such an assumption may easily be justified on biological grounds, since bone cells have their own duplication time, which is expected to remain the same both under ‘endogenous’ and ‘exogenous’ (i.e. hormonally induced) growth conditions.

**Figure 1:**
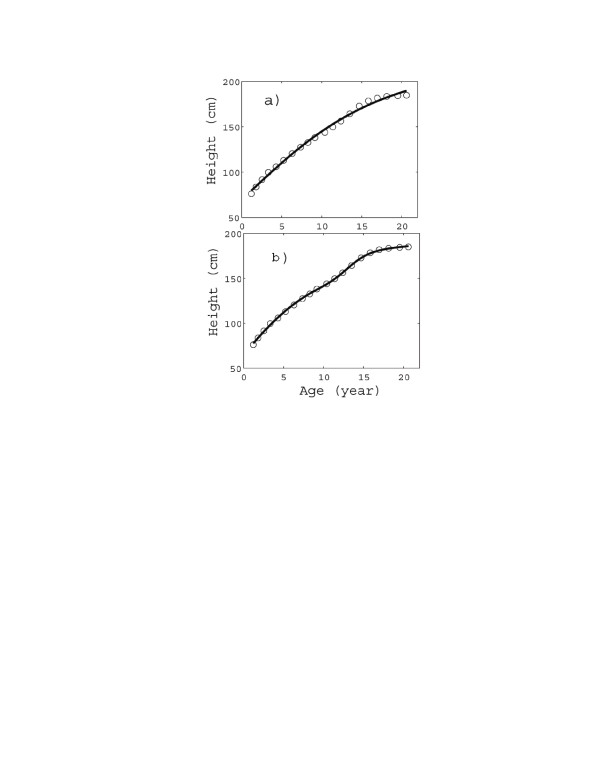
**Height vs. age data from dataset 1, British boys (1965): empty circles.** The solid curves represent the fitting of the data by means of the classes **(a)** U1, **(b)** (*U*1 + *F*1), respectively. The (*U*1 + *G*1) fitting curve is visually indistinguishable from (*U*1 + *F*1), and therefore is not shown separately. The fitting parameters and *R*^2^ values are reported in Table 2.

As discussed in the Background, two growth spurts are expected in the normal human development after birth, due to the stimulation of growth (GH) and sex (SH) hormones. However, as we shall see in the next section, only the latter has a significant impact on the growth chart morphology. Consequently, for the sake of reducing the number of model parameters, only the latter will be considered in the analysis of transversal datasets. However, a second spurt will also be included in the analysis of longitudinal datasets (concerning a single individual) when specific GH therapy is performed (see Figure 1 and Table 1). It should be remarked that there is, of course, also a spurt in the first year after birth. However, since perinatal growth has already been studied in [43], we do not consider it here and we restrict our analysis of human growth data to the time span from one year to maturity.

**Table 1:** Fitting parameter and *R*^2^ values corresponding to the longitudinal curve shown in Figure 1a (code IVS3)

		***U1***	***U1–H1***	***U1–F1***	***U1–F2***
ivs3		59.61	57.43	55.10	56.31
	*y*_0_(*cm*)	0.11	0.14	0.16	0.13
	*β*_0_(1/*yr*)	−0.09	−0.14	−0.15	−0.12
	*t*_*m*1_(*yr*)				2.60
	*y*_1_(*cm*)				0.01
	*β*_1_(1/*yr*)				−1.34
	*a*_1_(1/*yr*)				12.63
	*σ*_1_(*yr*)				0.02
	*t*_*m*2_(*yr*)		14.87	14.63	14.89
	*y*_2_(*cm*)		12.45	17.42	15.81
	*β*_2_(1/*yr*)		−0.11	−0.84	−2.38
	*σ*_2_(*yr*)			1.18	0.93
	*R*^2^	0.991	0.996	0.998	0.999

## Results

The formalism of the previous sections has been applied to the analysis of the following transversal datasets, representing statistical height vs. age (from 1 to 19 years) in large, well defined populations:

1. the “historical” growth charts published by [3] and based on the London County Council survey updated to 1965 [45,46]. In this survey a random sample of London schoolchildren was taken, including approximately 1,000 boys at each year of age;

2. the growth charts of British girls in 1990, recently re-published by Cole [47-49]. All measurements were made between 1978 and 1990 and included a total of over 25,000 individuals;

3. the growth charts of Romano et al. [50], referring to Noonan boys and girls, based on the results of the National Cooperative Growth Study (which includes a large number of Noonan children). Noonan syndrome is a genetically-based pathology whose incidence in the general population is estimated between 1:1,000 and 1:2,500 [51]. It is a clinically heterogeneous disorder predominantly characterized by dysmorphic facial features, congenital heart disease, proportionate post-natally short stature and other deformities [52].

Such data are normally used to define a “median” growth curve, which is the one considered in the following, and the percentiles, which are useful to pediatricians in order to establish threshold levels for underdevelopment.

**Figure 2:**
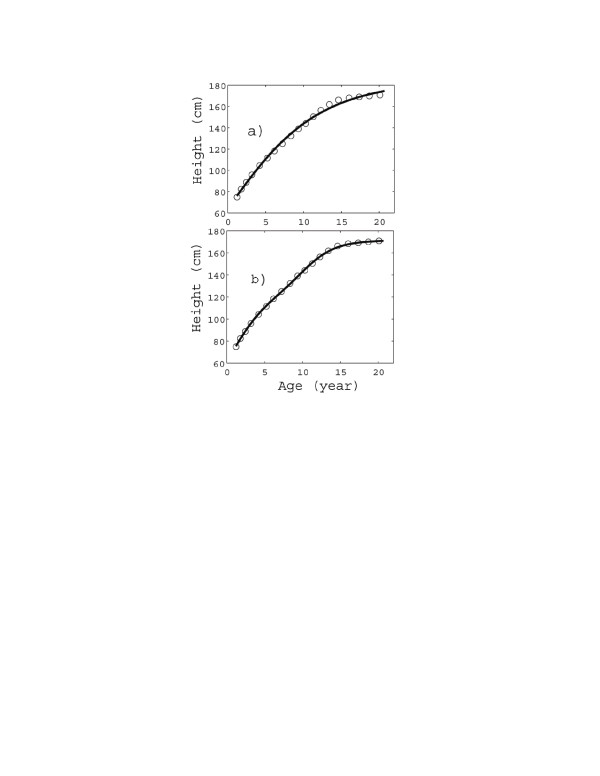
**Height vs. age data from dataset 2, British girls (1990): empty circles.** The solid curves represent the fitting of the data by means of the classes **(a)** U1, **(b)** (*U*1 + *F*1), respectively. The (*U*1 + *G*1) fitting curve is visually indistinguishable from (*U*1 + *F*1), and therefore is not shown separately. The fitting parameters and *R*^2^ values are reported in Table 2.

Figure 2 reports dataset 1 (British boys) and the corresponding fitting curves, based on the classes U1 and (*U*1 + *F*1); the (*U*1 + *G*1) curve coincides visually with the (*U*1 + *F*1) curve and is therefore omitted. It is clear from Figure 2a that the U1 curve is adequate for predicting the overall growth, but not the “structure”, which is quite conspicuous in the observational dataset at about 13 years. Such a structure is easily noticeable in the (*U*1 + *F*1) or (*U*1 + *G*1) curve (Figure 2b). In fact the quality of the fit in Figure 2c is truly remarkable, and well supported by the *R*^*2*^ values (see Table 2).

**Table 2:** Fitting parameters and *R*^2^ values corresponding to the curves shown in Figures 2, 3, 4, 5, 6 with reference to the three PUN classes *U*1, (*U*1 + *H*1) and (*U*1 + *F*1)

		***U1***	***U1 + H1***	***U1 + F1***
Boys (1965)	*y*_0_(*cm*)	69.67	66.71	65.06
	*a*_0_(1/*yr*)	0.12	0.14	0.16
	*β*_0_(1/*yr*)	-0.1	−0.14	−0.17
	*t*_*m*_(*yr*)		13.51	12.75
	*y*_1_(*cm*)		10.03	17.76
	*β*_1_(1/*yr*)		−0.53	−0.59
	*σ*(*yr*)			2.00
	*R*^2^	0.995	0.998	0.999
Girls (1990)	*y*_0_(*cm*)	64.14	62.91	60.65
	*a*_0_(1/*yr*)	0.15	0.1672	0.21
	*β*_0_(1/*yr*)	−0.15	−0.1696	−0.25
	*t*_*m*_(*yr*)		9.3377	9.37
	*y*_1_(*cm*)		4.3249	17.99
	*β*_1_(1/*yr*)		−0.1838	−0.38
	*σ*(*yr*)			3.21
	*R*^2^	0.995	0.997	0.999
Noonan boys	*y*_0_(*cm*)	67.58	65.34	62.22
	*a*_0_(1/*yr*)	0.09	0.1	0.13
	*β*_0_(1/*yr*)	−0.07	−0.1	−0.15
	*t*_*m*_(*yr*)		11	12.83
	*y*_1_(*cm*)		2.81	15.57
	*β*_1_(1/*yr*)		−0.08	−0.43
	*σ*(*yr*)			2.29
	*R*^2^	0.995	0.994	0.998
Noonan girls	*y*_0_(*cm*)	60.02	58.4	57.56
	*a*_0_(1/*yr*)	0.12	0.14	0.15
	*β*_0_(1/*yr*)	−0.12	−0.15	−0.16
	*t*_*m*_(*yr*)		12	11.79
	*y*_1_(*cm*)		5.83	9.26
	*β*_1_(1/*yr*)		−0.67	−0.93
	*σ*(*yr*)			1.28
	*R*^2^	0.995	0.998	0.998

**Figure 3:**
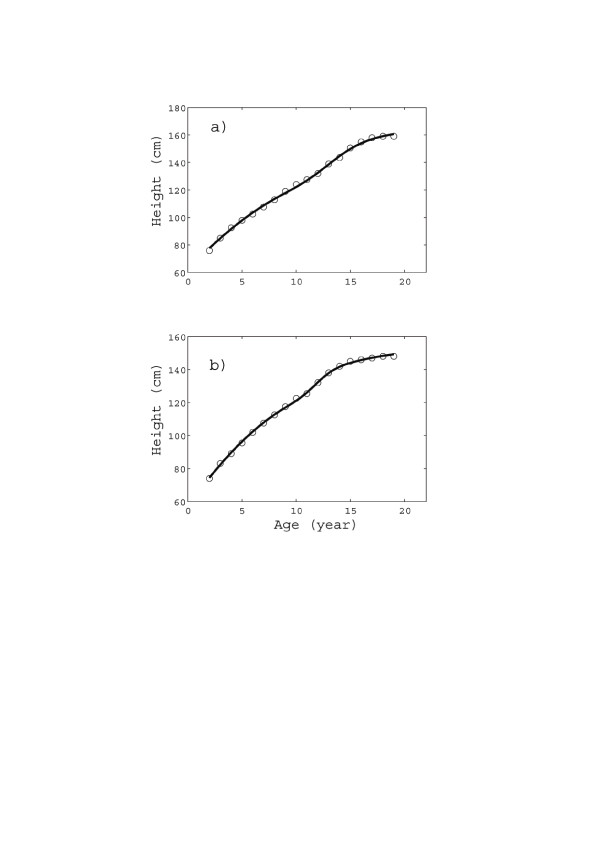
**Height vs. age from dataset 3 (Noonan children): (a) boys, (b) girls.** Only the fitting by means of the (*U*1 + *F*1) class is reported for brevity. The fitting parameters and *R*^2^ values are reported in Table 2.

Similar considerations hold also for the dataset 2 (British girls): see Figure 3. The same conclusions can be drawn, i.e. that U1 can predict only the overall pattern of growth, while (*U*1 + *F*1) also reproduce the “structure” of the curve. The two curves (*U*1 + *G*1) and (*U*1 + *F*1) are again almost indistinguishable. Thus the latter is preferable, since it requires one less parameter.

**Figure 4:**
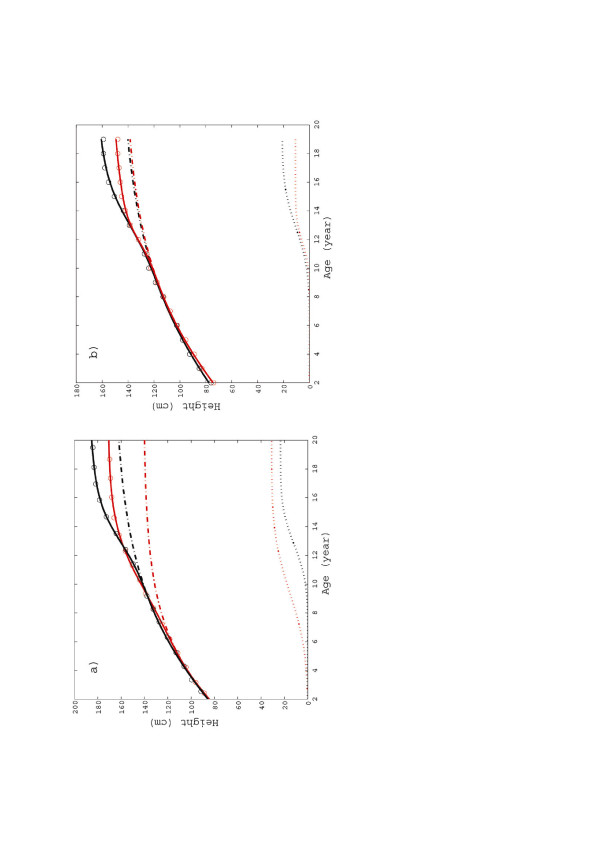
**Decomposition of the (*U*1 + *F*1) fitting curve (continuous lines) into two parts: U1 (dashed-dotted lines) and “spurt” (dotted lines).(a)** general population: boys (black) and girls (red on-line). **(b)** Noonan boys (black) and girls (red on-line).

Figure 4 shows the fitting of dataset 3 (Noonan boys and girls) by the (*U*1 + *F*1) PUN class. The quality of the (*U*1 + *F*1) fit is excellent also in the case of Figure 4.

**Figure 5:**
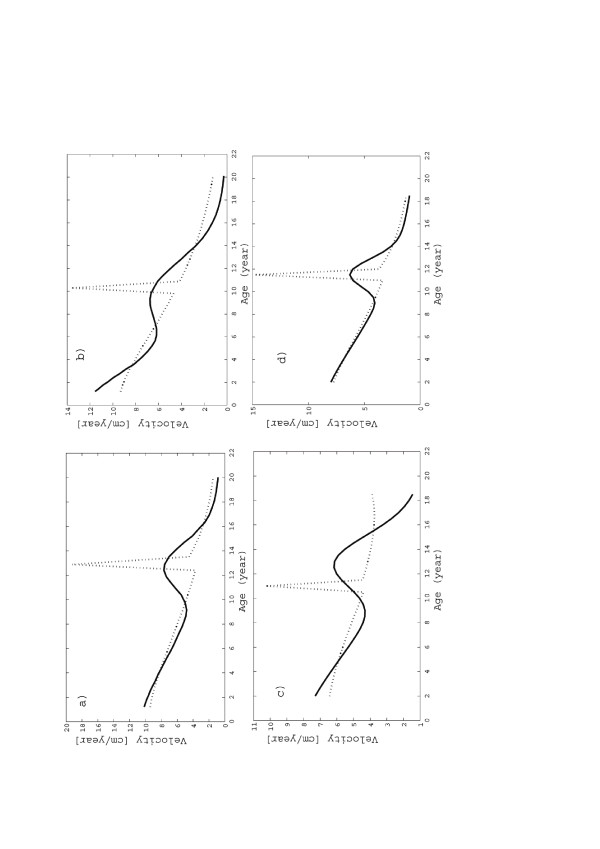
**Growth velocity for (a) general population: boys, (b) general population: girls, (c) Noonan boys, (d) Noonan girls.** The solid lines correspond to (*U*1 + *F*1) fitting, while the dotted lines refer to U1.

It may be interesting to show (see Figure 5) how the “structure” in Figures 2, 3, 4 originates as a result of the spurts, as discussed in the previous Section. Figure 5 shows how the plots of Figures 2b, 3b and 4b may be decomposed in the *U1* fitting curve plus the “spurts” (which are separately plotted in the lower parts of Figure 5a and b as dotted lines). The two figures refer to “normal” (general population) and Noonan children, respectively, and consider separately boys and girls in both cases. One can see that both the pre-spurt U1 growth and the spurt are almost identical for boys and girls (actually the spurts are larger for the latter). Nevertheless the height at maturity is statistically lower for normal girls, since for them puberty, hence saturation of the U1 growth and onset of the spurt, happens on the average almost three years earlier. However, in comparing the data for boys and girls it is necessary to recall that the two datasets refer to different years (1965 vs. 1990), i.e. they are separated by 25 years of socio-economic development (and consequent impact on child growth). It is interesting to observe that, contrary to the case of normal boys and girls, the time of onset of the spurts for Noonan children seems to be gender-independent.

**Figure 6:**
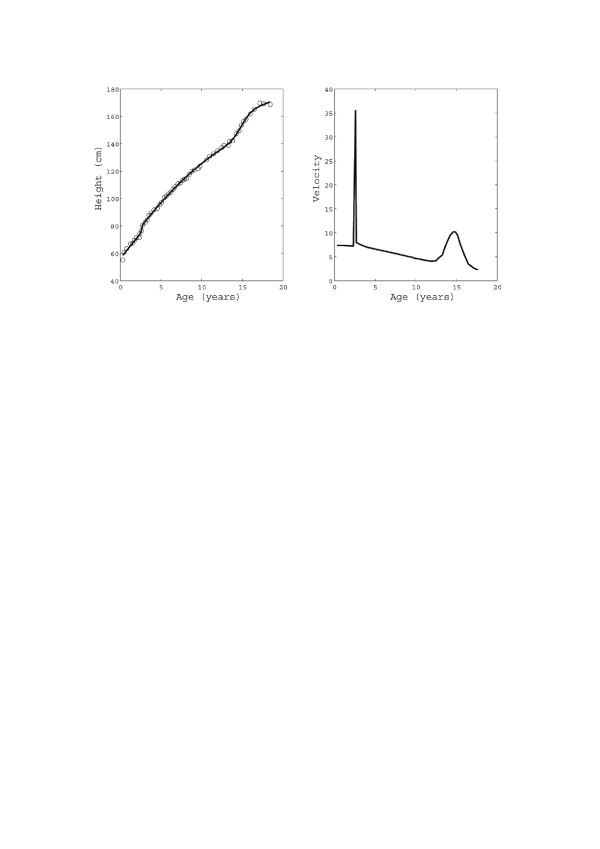
**(a) Height vs. age data for an individual male denoted by the code IVS3 **[44]** and corresponding (*U*1 + *F*2) fitting. (b)** Corresponding velocity.

It may be instructive to plot the growth velocity, defined as

(10) $$v(t)=\frac{dy(t)}{dt}=a(y(t))y(t)$$

vs. time (see Figure 6). The solid lines in the four subplots of Figure 6 (i.e. general population and Noonan, boys and girls) exhibit a smooth but relevant change in the velocity pattern in the (*U*1 + *F*1) curves at the onset of puberty. By comparison, the U1 curves (dotted lines) have very high and narrow peaks at the inception of the spurts, which are not consistent with natural development and even less with the statistical nature of the data.

Finally it can be observed that *β*_1_ is larger (in absolute value) for boys (−0.59) than for girls (−0.38) in the general population, while *β*_1_ for Noonan girls (−0.93) is larger than for Noonan boys (−0.43). Something similar happens with *σ*.

All the datasets analysed up to here are transversal, as stated. However, in order to evaluate the effect of Growth Hormone (GH) administration therapy to selected cases of critically short stature in infancy, “longitudinal” curves should be investigated. As an example, the dataset referring to a single young male, denoted by the code IVS3 in ref. [44], is analysed in Figure 1: it shows the two spurts, the first corresponding to the therapeutic administration of GH and the second to prepuberal growth (Table 1 reports the corresponding parameters and *R*^2^ values). In this case, as well as in similar ones, idiopathic short stature is corrected by the therapeutic administration of GH, which induces an additional ‘artificial’ spurt. A (U1 + F2) model is therefore required to account for the two spurts.

## Conclusions

Growth charts represent a very useful reference tool for pediatricians to monitor the growth of individual children (i.e. size, rate of growth and effects of an eventual treatment). They are transversal in the sense that they yield avarage values (divided into percentiles) over large, well defined populations, e.g. boys (or girls) in a given country and time frame. Several, mostly heuristic, formulations have been proposed in order to model the growth, i.e. to fit e.g. the median growth curve (out of the charts) from early childhood to maturity. In this contribution we have presented an alternative approach, based on the formalism of the Phenomenological Universalities (PUN) [33,34], which represent a new, completely general and interdisciplinary methodology.

As a result, we have found that the main PUN class studied to date, i.e. U1 and U2 [36-38,41], can only predict the overall human growth pattern. For a more realistic description it is necessary to add to it one or more “spurts”, as also suggested by other authors [49] and well justified on biological grounds. Consequently a new PUN class (*UN* + *FM*) has been developed and shown to provide excellent agreement with some standard auxological datasets. Its formalism can be suitably related to the biological description of bone plate growth under selective hormonal stimulation of the bone epiphysis. In other words, the additional increase of stature is the “macroscopic” response to a well defined biological signal.

The accuracy of the fitting (see Figures 1a, 2c, 3c, and 4) is very important not only “per se”, but also because it adds a real significance to the model parameters. E.g., by comparing Figures 2c and 3^2^c, we not only find that boys grow much later but faster than girls (as is well known), but we are also able to quantify the difference: growth spurt inception time (13 *yr* vs. 9 *yr*) and duration (2 *yr* vs. 3.2 *yr*) and total accretion due to the spurt (almost the same: 17.7 *cm* vs. 18 *cm*).

Likewise we may quantify the differences between the growth patterns of healthy vs. growth impaired children (e.g. Noonan [50-52]). E.g., as we can see in Table 2, the time of pubertal growth onset is quite different in Noonan vs. control girls (about two years later for the latter), while it is approximately the same for males. Likewise for the pubertal growth duration. As a consequence the difference in “added” stature is relatively small for boys (about 2 *cm*), while it is dramatic for girls (about 8 *cm*!). This suggests (even if other effects should also be considered, such as the time of reference for measurements) that pubertal growth is much more severely affected by Noonan syndrome in females than in males.

**Figure 7:**
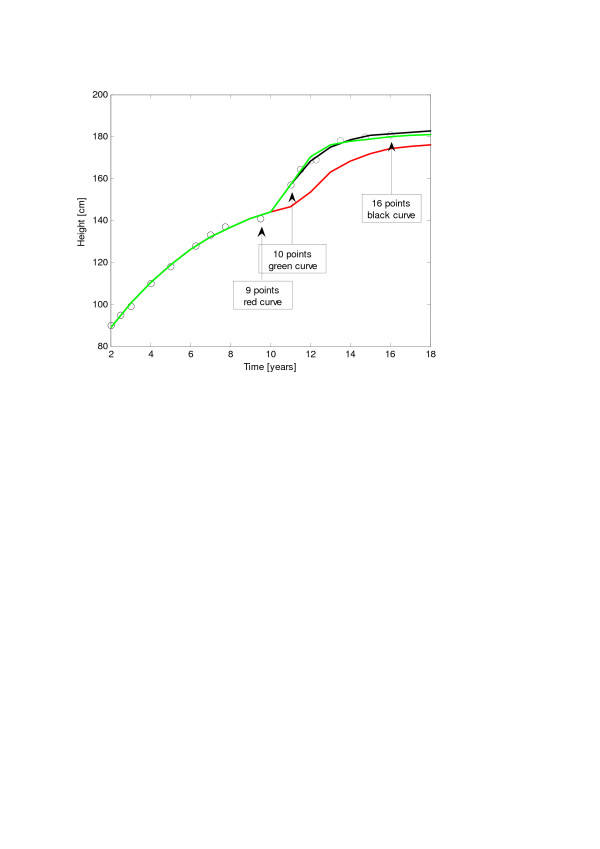
**Instance of longitudinal growth curve relative to a normal boy fitted by means of the (*U*1 + *F*1) model with a different number of datapoints: 9 points (red curve), 10 points (green curve) and 16 points (black).**

We have also presented the result of one case of idiopathic short stature treated by the administration of GH. Correspondingly our computer program has revealed the appearance at about 3 years of age of a very short spurt (see Figure 1a), caused by a very narrow peak in the growth velocity (Figure 1b). Although a more careful analysis would be required to correlate the dosage of GH and its incremental effect on bone length, we believe that our model could be further refined and be used as a “clinical simulator” for the optimization of GH therapies. This approach could eventually help pediatricians and endocrinologists to optimise the clinical protocol for each specific growth problems. As an example we have analyzed a longitudinal growth curve relative to a normal boy. We tried a sort of blind test for the model. We took the first 9 points of the curve (before the spurt) and fitted (red curve), using, as an initial guess of the model, the parameters relative to the growth of the normal boys (Table 2, U1 + F1), in order to forecast the further growth of the boy. Then we repeated the procedure with 10 points (one point after the spurt, green curve) and with all the 17 points (black curve). It is clear from Figure 7 that the information about the localization in time and intensity of the “spurt” is necessary to yield a good prediction. In fact, in this particular case, the pubertal development happened earlier with respect to the mean value, which, of course, could not be forecast by the model.

To conclude, the formalism presented in this contribution may also be applied to the analysis of other datasets of auxological interest, e.g. referring to variables such as the Body Mass Index (BMI): see e.g. [47]. It may also be used for the systemic analysis of two or more variables assembled as a complex or vectorial quantity [39]. In this case the goal is not only to investigate their time evolution, but also the degree of correlation and mutual dependence. As an example it would be instructive to study how mass growth follows stature in terms of relative increment of the various body components occurring differently with age, sex and life style.

## Competing interests

The authors have no competing interests to disclose.

## Authors’ contributions

CG and PPD derived the model and wrote the first draft of the manuscript, AG performed the calculations and the numerical work. The model concept was devised by ASG, CG, PPD and DAI. All authors have read and approved the final manuscript.
